# The vascular endothelial growth factor as a candidate biomarker of systemic lupus erythematosus: a GRADE-assessed systematic review and meta-analysis

**DOI:** 10.1007/s10238-024-01487-w

**Published:** 2024-09-11

**Authors:** Arduino A. Mangoni, Angelo Zinellu

**Affiliations:** 1 Discipline of Clinical Pharmacology, College of Medicine and Public Health, Flinders University, Adelaide, Australia; 2 Department of Clinical Pharmacology, Flinders Medical Centre, Southern Adelaide Local Health Network, Adelaide, Australia; 3 Department of Biomedical Sciences, University of Sassari, Sassari, Italy; 4 Department of Clinical Pharmacology, College of Medicine and Public Health, Flinders University and Flinders Medical Centre, Bedford Park, SA 5042 Australia

**Keywords:** Vascular endothelial growth factor, VEGF, Systemic lupus erythematosus, SLE, Biomarkers, Endothelial damage, Active disease, Organ dysfunction

## Abstract

There is an ongoing search for novel biomarkers of endothelial damage, active disease, and organ dysfunction in systemic lupus erythematosus (SLE). We investigated the role of the vascular endothelial growth factor (VEGF) as a candidate biomarker by conducting a systematic review and meta-analysis of studies examining VEGF concentrations in SLE patients and healthy controls. We searched electronic databases (PubMed, Scopus, and Web of Science) from inception to 31 May 2024 (inclusion criteria: VEGF measurement in SLE patients and healthy controls and SLE patients with and without active disease or specific organ dysfunction in case–control studies, recruitment of adult participants, and availability of the full text in the English language; exclusion criteria: non-case–control studies, participants under 18 years, articles reporting duplicate or irrelevant data, and animal studies). We assessed the risk of bias and the certainty of evidence using the JBI Critical Appraisal Checklist and GRADE, respectively (PROSPERO registration number: CRD42024561636). Circulating VEGF concentrations were significantly higher in SLE patients than in controls (22 studies; standardised mean difference, SMD = 0.71, 95% CI 0.44 to 0.98, *p* < 0.001; low certainty of evidence). In SLE patients, VEGF concentrations were significantly higher in those with active disease (six studies; SMD = 1.10, 95% CI 0.27 to 1.92, *p* = 0.009; very low certainty of evidence) and lupus nephritis (four studies; SMD = 0.80, 95% CI 0.03 to 1.57, *p* = 0.042; very low certainty of evidence). Only one study reported VEGF concentrations in SLE patients with and without pulmonary arterial hypertension. The effect size of the differences in VEGF concentrations between SLE patients and controls was not associated with disease duration, use of glucocorticoids and immunosuppressors, biological matrix assessed, or analytical method used. However, it was significantly associated with the study’s geographical location. The evidence was limited by the high but partially explainable heterogeneity and the presence of publication bias which was addressed with the “trim-and-fill” method (SLE presence), the high but partially explainable heterogeneity and lack of assessment of publication bias because of the limited study number (active disease), and the limited study number preventing the identification of sources of heterogeneity, sensitivity analysis, and assessment of publication bias (lupus nephritis). Our results highlight VEGF’s potential role as a SLE biomarker and the need for further research, also given the aforementioned limitations, investigating VEGF concentrations in a wide range of SLE patient subgroups.

**Supplementary Information:**

The online version contains supplementary material available at 10.1007/s10238-024-01487-w.

## Introduction

Systemic lupus erythematosus (SLE) is an autoimmune condition characterised by the abnormal production of antinuclear antibodies, immune complex deposition, and chronic inflammation affecting various organs and systems [1, 2]. Whilst the dysregulation of immune and inflammatory pathways are critical factors in the pathophysiology of SLE, an increasing body of research suggests the involvement of other molecular and cellular pathways. A better understanding of such pathways and identifying novel candidate biomarkers might enhance diagnosis and monitoring in this patient group [1–3].

Several epidemiological studies have shown that cardiovascular disease is one of the leading causes of death in SLE, together with renal disease and infection [4–7]. Notably, the prevalence and impact of traditional risk factors, e.g. hypertension, diabetes, and dyslipidaemia, on cardiovascular mortality have been shown to be similar in SLE patients and the general population [8–10]. These observations suggest that alternative pathways, e.g. inflammation and immune activation, may adversely affect endothelial function [11, 12], favouring the onset and progression of atherosclerosis, and ultimately accounting for the reported association between SLE and cardiovascular disease [13]. Atherosclerosis, the critical driver of the clinical manifestations of cardiovascular disease, e.g. myocardial infarction and stroke, is a chronic inflammatory condition that develops due to structural and functional alterations affecting the endothelial cell layer, leading to endothelial damage [14, 15]. Studies conducted over the last 20 years have reported that patients with SLE have an increased risk of endothelial dysfunction as a surrogate marker of subclinical and overt atherosclerosis [16].

The vascular endothelial growth factor-A (VEGF) is one of the gene products of the vascular endothelial growth factor family [17]. Following its binding to its primary receptors, VEGFR-1 and VEGFR-2, VEGF regulates the differentiation of endothelial progenitor cells, endothelial cell function, and angiogenesis [18]. However, the pathophysiological role of VEGF in SLE is controversial. Experimental studies have reported a reduced expression of VEGF in SLE, with a consequent reduction in the number of endothelial progenitor cells and alteration in their physiological functions [19–21]. VEGF suppression in SLE has been shown to be secondary to the upregulation of the genes encoding the pro-inflammatory cytokine, interferon alpha [19]. By contrast, studies conducted in SLE patients have shown increased circulating concentrations of VEGF. Such elevations were associated with the upregulation of several pro-inflammatory cytokines, e.g. interleukin-6 and interleukin-8 [22].

Given the contrasting results of in vitro and in vivo studies and the pathophysiological role of dysregulated angiogenesis and inflammation in the occurrence of organ dysfunction in this patient group, e.g. lupus nephritis and pulmonary arterial hypertension [23, 24], we critically appraised the available evidence regarding the association between VEGF and SLE. Specifically, we conducted a systematic review and meta-analysis of studies investigating circulating VEGF concentrations in SLE patients and healthy controls and SLE patients with different disease activity and organ dysfunction. Where possible, we also assessed possible associations between the magnitude of the between-group differences in VEGF concentrations and several study and patient characteristics.

## Materials and methods

### Literature search and study selection

We conducted a systematic literature search in the electronic databases PubMed, Scopus, and Web of Science from inception to 31 May 2024, using the terms “systemic lupus erythematosus” OR “SLE” AND “VEGF” OR “vascular endothelial growth factor”. (The details of the search strategy in each database are described in Supplementary Table 1.) Two investigators independently screened each abstract. If relevant, they independently reviewed the full text of each article. The inclusion criteria were: (1) the measurement of VEGF concentrations in SLE patients and healthy controls and in SLE patients with and without active disease or specific organ dysfunction in case–control studies, (2) the recruitment of adult participants, and (3) the availability of the full text of the publication in the English language. Exclusion criteria were (1) studies with a non-case–control design, (2) inclusion of participants under 18 years, (3) articles reporting duplicate or irrelevant data, and (4) animal studies. The investigators also independently hand-searched the references of the retrieved articles for additional studies.

The investigators independently extracted the following variables from each article: publication year, first author details, country and continent where the study was conducted, number of participants, age, male-to-female ratio, mean disease duration, VEGF concentrations, sample matrix assessed and analytical method used, use of glucocorticoids and immunosuppressants, and presence of active disease and specific organ dysfunction (e.g. lupus nephritis and pulmonary arterial hypertension).

The risk of bias in each article was evaluated using the Joanna Briggs Institute (JBI) Critical Appraisal Checklist for analytical studies [25]. The certainty of evidence for each endpoint was evaluated using the Grades of Recommendation, Assessment, Development, and Evaluation (GRADE) Working Group system [26]. The Systematic Reviews and Meta-Analyses (PRISMA) 2020 statement was fully adhered to in the preparation of the manuscript (Supplementary Table 1) [27]. We registered the study protocol in an international repository (PROSPERO registration number: CRD42024561636).

### Statistical analysis

We generated forest plots of standardised mean differences (SMDs) and 95% confidence intervals (CIs) to investigate between-group differences in VEGF concentrations (*p* < 0.05 for statistical significance). If required, data were extracted from graphs using the Graph Data Extractor software (San Diego, CA, USA). Furthermore, means and standard deviations were extrapolated from medians and interquartile or full ranges, as previously reported [28]. The heterogeneity of the SMD across studies was evaluated using the *Q* statistic (*p* < 0.10 for statistical significance) and classified as low (I^2^ ≤ 25%), moderate (25% < I^2^ < 75%), and high (I^2^ ≥ 75%) [29, 30]. A random-effects model based on the inverse-variance method was used in the presence of high heterogeneity. Sensitivity analysis and assessment of publication bias were conducted using standard procedures [31–34].

We conducted univariate meta-regression and subgroup analyses to investigate associations between the effect size and the following parameters: year of publication, country and continent where the study was conducted, sample size, age, male-to-female ratio, mean disease duration, sample matrix, analytical method used, presence of active disease and organ dysfunction, and use of glucocorticoids or immunosuppressants. Statistical analyses were performed using Stata 14 (Stata Corp., College Station, TX, USA).

## Results

**Fig. 1:**
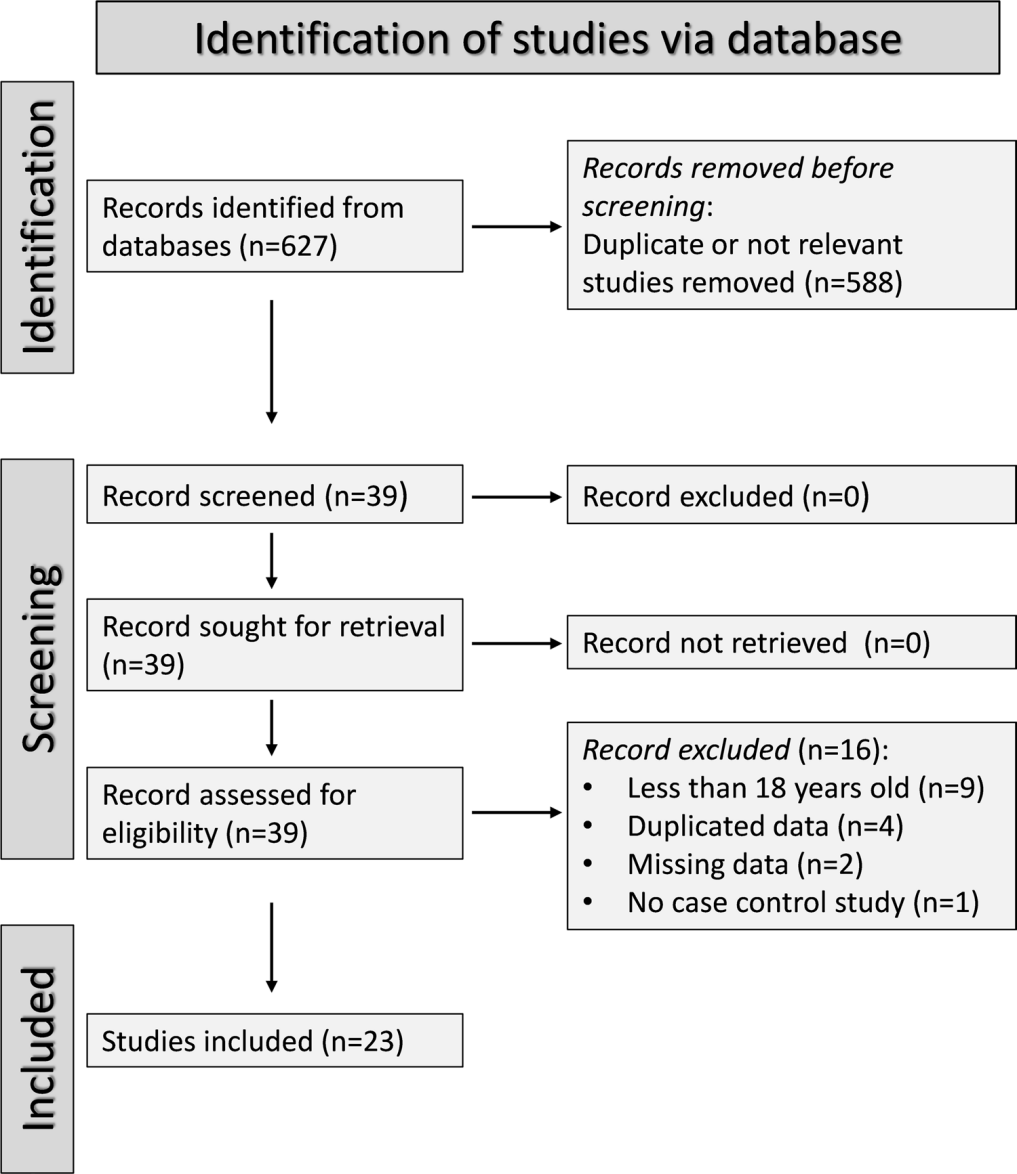
PRISMA 2020 flow diagram of study screening and selection

Figure 1 illustrates the flow chart of the screening process and study selection. From 627 articles initially identified, we excluded 588 following the first screening step because they were either duplicates or irrelevant. A review of the full text of the remaining 39 articles led to the further exclusion of nine studies including participants under 18 years, four because of duplicate data, two because of missing data, and one because it was not case–control design. Therefore, 23 studies were selected for analysis [35–57] (Tables 1, 2 and 3). The risk of bias (Supplementary Table 2) was low in 14 studies [37, 42, 43, 47–57] and moderate in the remaining nine [35, 36, 38–41, 44–46]. The initial level of the certainty of evidence was ranked as low (level 2) given the cross-sectional nature of the selected studies.

**Table 1:** Characteristics of studies investigating vascular endothelial growth factor in patients with systemic lupus erythematosus and healthy controls

Study	Sample matrix	Controls				Patients with systemic lupus erythematosus				
		*n*	Age (years)	M/F	VEGF (Mean ± SD)	*n*	Age (years)	M/F	VEGF (Mean ± SD)	MDD (years)
Harada et al. 1998, Japan [35]	S	10	Matched	4/6	74.1 ± 31.9	10	Matched	1/9	97.8 ± 52.5	NR
Kikuchi et al. 1998, Japan [36]	S	20	47.5	4/16	184 ± 62	17	44.8	3/14	242 ± 109	NR
Robak et al. 2000, Poland [37]	S	20	45.8	3/17	124.7 ± 59.7	60	44.8	5/55	234.2 ± 209.9	9.01
Navarro et al. 2002, Mexico [38]	P	24	29.2	5/19	88.6 ± 78.9	28	36.6	4/24	121.6 ± 83.6	NR
Kuryliszyn–Moskal et al. 2007, Poland [39]	S	30	Matched	Matched	148 ± 40	47	40.8	3/44	228 ± 158	8.1
Tanaseanu et al. (a) 2007, Romania [40]	P	10	NR	NR	330.3 ± 84.16	15	35	3/12	744.2 ± 425.1	NR
Tanaseanu et al. (b) 2007, Romania [40]	P	10	NR	NR	330.3 ± 84.16	15	45	4/11	1023.1 ± 259.1	NR
Ciprandi et al. 2008, Italy [41]	S	40	43	7/33	462.6 ± 275	40	41.95	0/40	586 ± 450	NR
Rho et al. 2008, USA [42]	S	78	40.5	11/67	38.5 ± 52.6	109	40.2	9/100	46.6 ± 47.7	NR
Colombo et al. 2009, Italy[43]	P	80	40.1	0/80	120.7 ± 118.4	80	42.6	0/80	307.9 ± 292.2	NR
Hrycek et al. 2009, Poland [44]	S	24	51	0/24	2869 ± 1625	48	47	0/48	3252 ± 1732	NR
Robak et al. 2013, Poland [45]	S	20	Matched	3/17	202.5 ± 117.6	60	39	5/55	431.9 ± 311.6	5.5
Koca et al. 2013, Turkey [46]	NR	28	42.5	6/22	330.9 ± 195.6	23	37.9	2/21	210.2 ± 175.3	5.1
Zhou et al. 2014, China [47]	S	28	37.82	6/22	47.29 ± 52.62	54	36.81	4/50	91.47 ± 108.67	NR
Barbulescu et al. 2015, Romania [48]	S	17	NR	1/16	31.84 ± 11.74	18	45	2/16	68.99 ± 71.06	8
Ghazali et al. 2017, Malaysia [49]	S	26	33.19	0/26	343 ± 146.82	92	30.43	2/90	494.89 ± 352.87	3.2
Willis et al. (a) 2017, USA [50]	S	30	43.5	5/25	98 ± 66	267	47.6	15/252	206 ± 189	16.9
Willis et al. (b) 2017, USA[50]	S	30	43.5	5/25	98 ± 66	45	44	1/44	194 ± 167	6.8
Idborg et al. 2018, Sweden [52]	P	322	47.4	26/296	59 ± 30	437	46.5	35/402	73 ± 79	11.4
Zhao et al. 2018, China [53]	P	30	30	4/26	68 ± 27	67	33.4	9/58	113 ± 61	4.2
El-Gazzar et al. 2019, Egypt [55]	S	33	Matched	0/33	76.5 ± 33.01	84	29.03	0/84	417.1 ± 410.4	5.2
Barraclough et al. 2019, UK [54]	NR	30	40.25	0/30	54.5 ± 84.8	36	35.17	2/34	73.2 ± 97	NR
Tokarska et al. 2020, Poland [56]	S	24	30.9	8/16	338 ± 258.2	36	49.6	4/32	421 ± 342.3	14.7
Ene et al. 2023, Romania [57]	S	60	41.7	10/50	118.6 ± 20.6	86	43	17/69	377.3 ± 88.3	5.7

Legend: MDD, mean disease duration; M/F, male-to-female ratio; NR, not reported; P, plasma; S, serum; VEGF, vascular endothelial growth factor. VEGF concentrations were expressed as pg/mL

**Table 2:** Characteristics of studies investigating vascular endothelial growth factor in patients with systemic lupus erythematosus with and without active disease

Study	Sample matrix	No active disease				Active disease				
		*n*	Age (years)	M/F	VEGF (Mean ± SD)	*n*	Age (years)	M/F	VEGF (Mean ± SD)	MDD (years)
Robak et al. 2000, Poland [37]	S	28	NR	NR	165.3 ± 153.4	32	NR	NR	300.8 ± 250.9	9.01
Kuryliszyn − Moskal et al. 2007, Poland [39]	S	28	NR	NR	153 ± 61	19	NR	NR	303 ± 211	8.1
Robak et al. 2013, Poland [45]	S	32	NR	NR	388.93 ± 295.12	28	NR	NR	469.42 ± 325.34	5.5
Zhou et al. 2014, China [47]	S	18	41.44	2/16	72.7 ± 39.5	36	34.5	2/34	100.87 ± 129.89	NR
Gao et al. 2018, China [51]	S	40	34.4	5/35	186.3 ± 11.5	40	34.3	6/34	266.1 ± 23.9	2.55
Idborg et al. 2018, Sweden [52]	P	115	NR	NR	67 ± 45	322	NR	NR	90 ± 60	11.4

Legend: MDD, mean disease duration; M/F, male-to-female ratio; NR, not reported; P, plasma; S, serum; VEGF, vascular endothelial growth factor. VEGF concentrations were expressed as pg/mL

**Table 3:** Characteristics of studies investigating vascular endothelial growth factor in patients with systemic lupus erythematosus with and without lupus nephritis

Study	Sample matrix	No lupus nephritis				Lupus nephritis				
		*n*	Age (years)	M/F	VEGF (Mean ± SD)	*n*	Age (years)	M/F	VEGF (Mean ± SD)	MDD (years)
Ghazali et al. 2017, Malaysia [49]	S	46	NR	NR	436.14 ± 410.44	46	NR	NR	553.65 ± 295.29	3.2
Idborg et al. 2018, Sweden [52]	P	257	NR	NR	80.7 ± 55.8	180	NR	NR	90.2 ± 63.8	11.4
Zhao et al. 2018, China [53]	P	35	34.3	5/30	80 ± 37	32	32.4	4/28	144 ± 82	4.2
Ene et al. 2023, Romania [57]	S	86	43	17/69	377.3 ± 88.3	73	44.1	11/62	541.5 ± 103.5	5.7

Legend: MDD, mean disease duration; M/F, male-to-female ratio; NR, not reported; P, plasma; S, serum; VEGF, vascular endothelial growth factor. VEGF concentrations were expressed as pg/mL

### Presence of systemic lupus erythematosus

As reported in Table 1, 22 studies, including 24 group comparators, investigated VEGF in 1,774 SLE patients (mean age 42 years, 93% females) and 1024 healthy controls (mean age 42 years, 89% females) [35–50, 52–57]. Twelve studies were conducted in Europe [37, 39–41, 43–45, 48, 52, 54, 56, 57], six in Asia [35, 36, 46, 47, 49, 53], three in America [38, 42, 50], and one in Africa [55]. VEGF was measured using an enzyme-linked immunosorbent assay in 18 studies [35–45, 47–49, 53, 55–57] and a platform for multi-analyte profiling in two studies [50, 52]. The remaining two studies provided no details regarding the assay used [46, 54]. Fifteen studies assessed serum [35–37, 39, 41, 42, 44, 45, 47–50, 55–57] and five plasma [38, 40, 43, 52, 53]. The remaining two studies provided no details regarding the biological matrix assessed [46, 54]. Disease duration was reported in 12 studies and ranged between 3.2 and 16.9 years [37, 39, 45, 46, 48–50, 52, 53, 55–57]. The risk of bias (Supplementary Table 2) was low in 13 studies [37, 42, 43, 47–50, 52–57] and moderate in the remaining nine [35, 36, 38–41, 44–46].

**Fig. 2:**
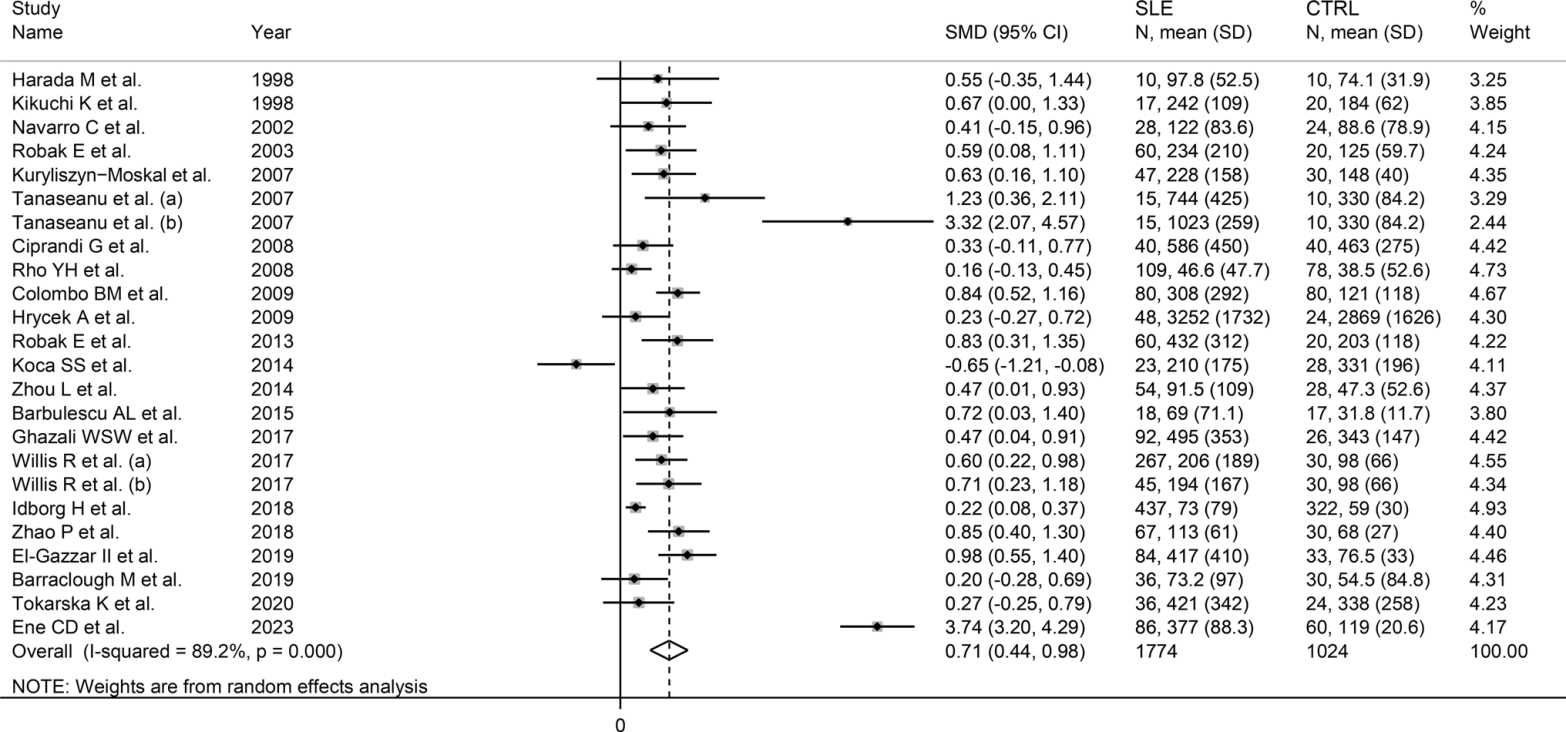
Forest plot of studies investigating vascular endothelial growth factor concentrations in patients with systemic lupus erythematosus and healthy controls

**Fig. 3:**
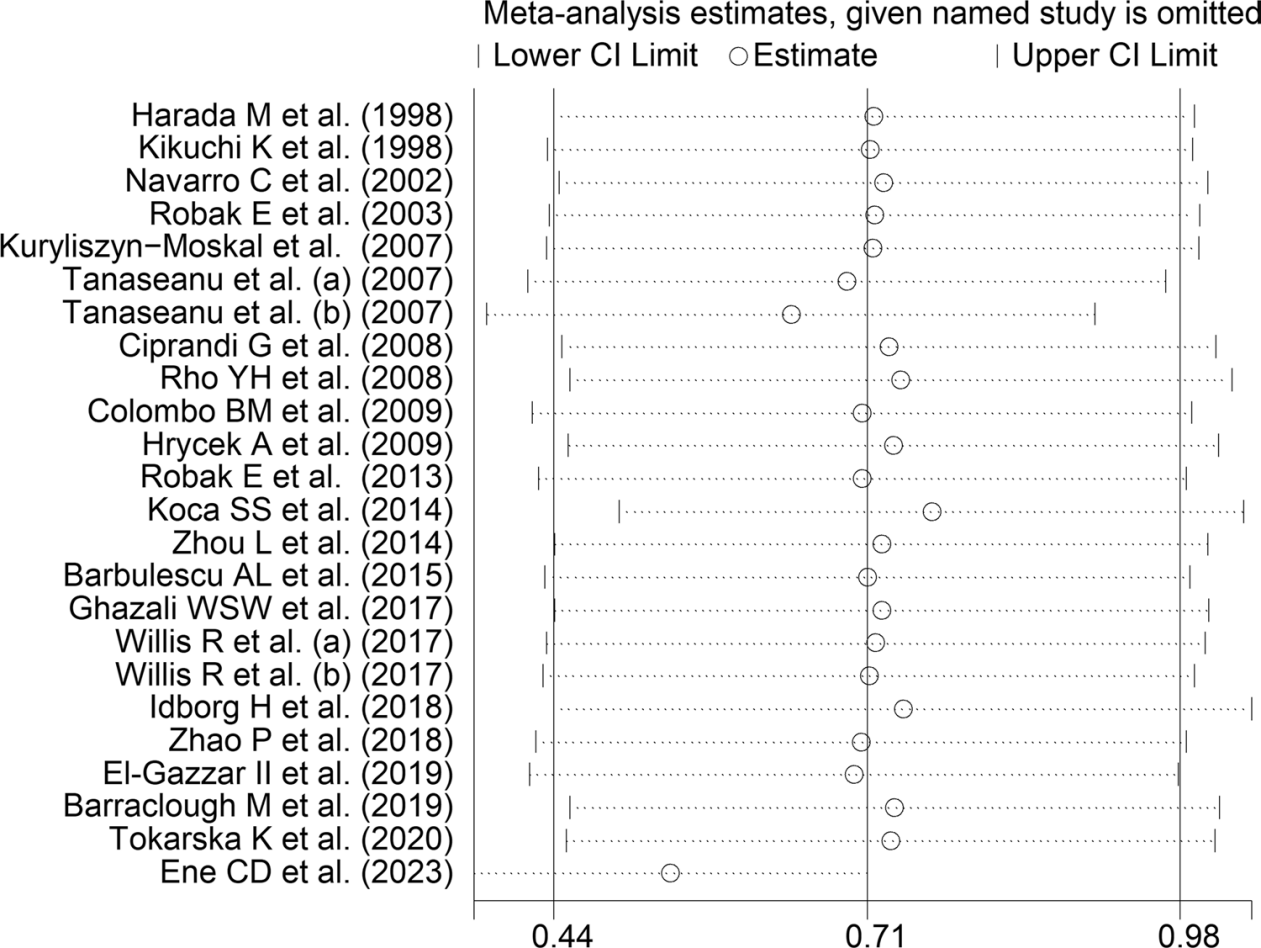
Sensitivity analysis of the association between vascular endothelial growth factor and systemic lupus erythematosus

**Fig. 4:**
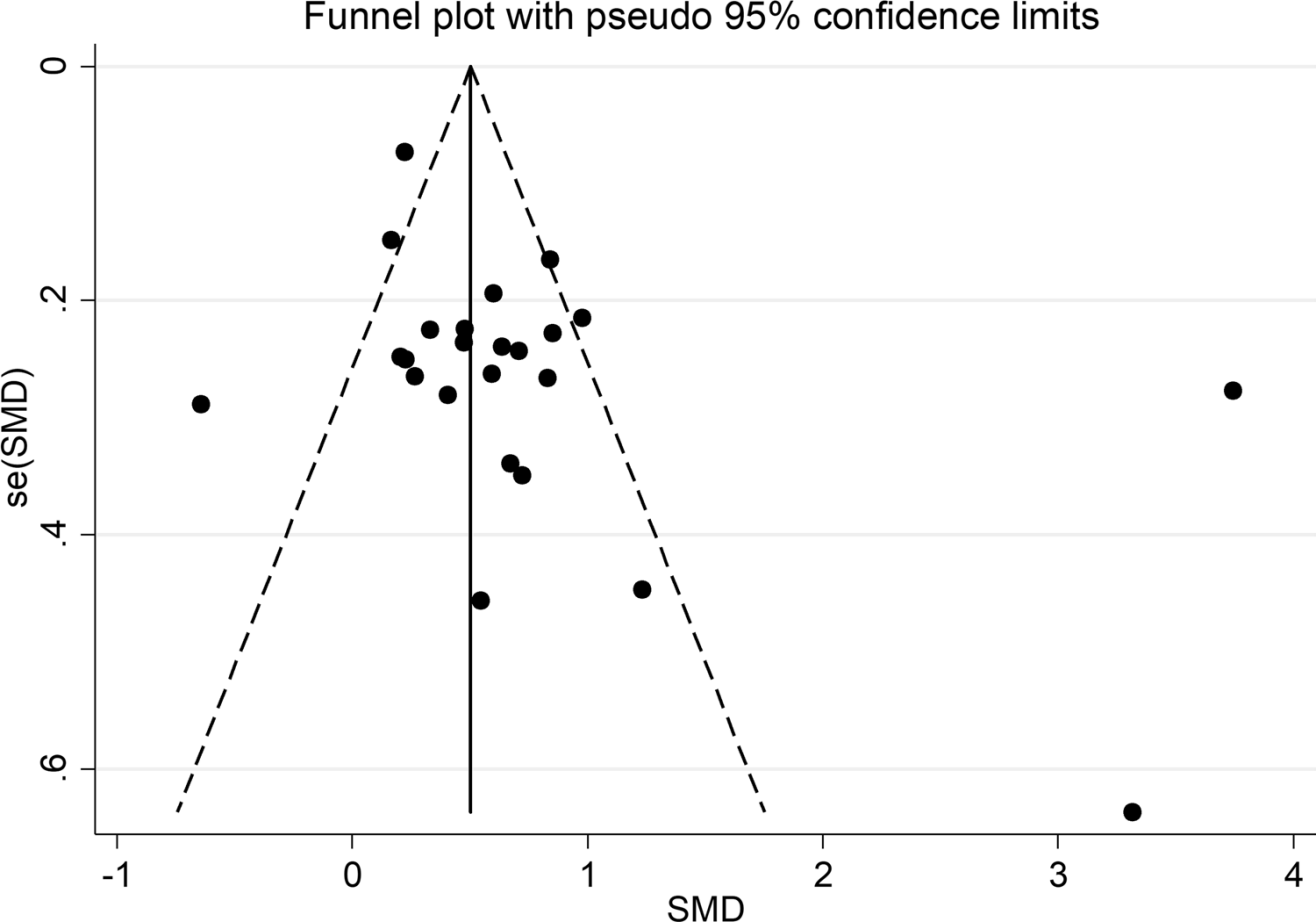
Funnel plot of studies investigating the association between vascular endothelial growth factor and systemic lupus erythematosus

The forest plot (Fig. 2) showed that SLE patients had significantly higher VEGF concentrations than controls (SMD = 0.71, 95% CI 0.44 to 0.98, *p* < 0.001; I^2^ = 89.2%, *p* < 0.001). The pooled SMD was stable in sensitivity analysis, ranging between 0.54 and 0.76 (Fig. 3). However, the funnel plot (Fig. 4) revealed the distortive effect of two studies [40, 47]. Their removal was associated with a reduced, yet still significant, effect size (SMD = 0.49, 95% CI 0.34 to 0.64, *p* < 0.001) and a lower between-study variance (I^2^ = 60.3%, *p* < 0.001).

**Fig. 5:**
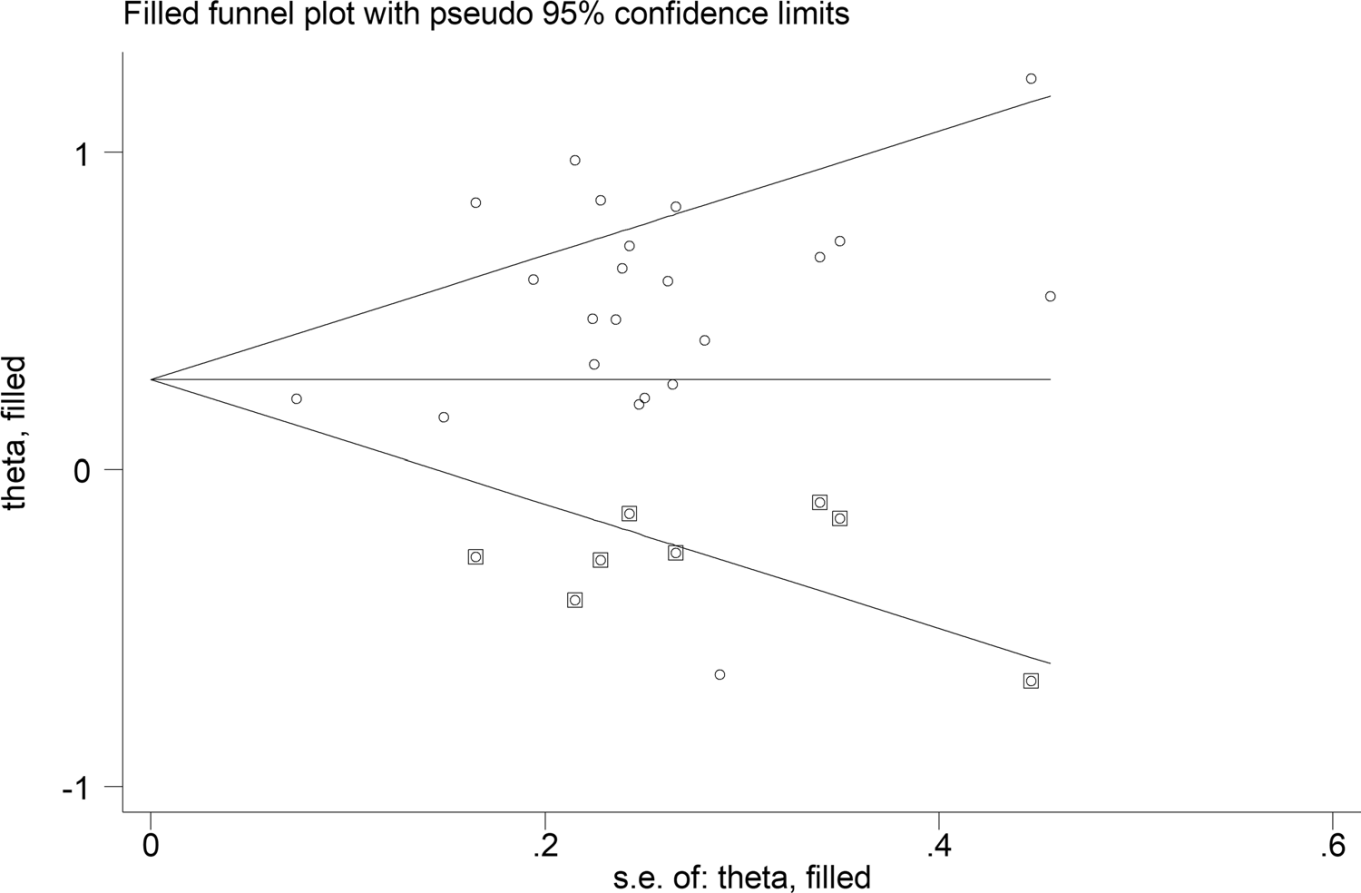
Funnel plot of studies investigating the association between vascular endothelial growth factor and systemic lupus erythematosus after “trimming and filling”. Dummy and genuine studies are represented by enclosed and free circles, respectively

There was significant publication bias according to Egger’s test (*p* = 0.065) but not Begg’s (*p* = 1.00). Eight missing studies to be added to the left side of the funnel plot to ensure symmetry were identified using the “trim-and-fill” (Fig. 5). The resulting effect size was further attenuated but still significant (SMD = 0.30, 95% CI 0.14 to 0.46, *p* < 0.001).

**Fig. 6:**
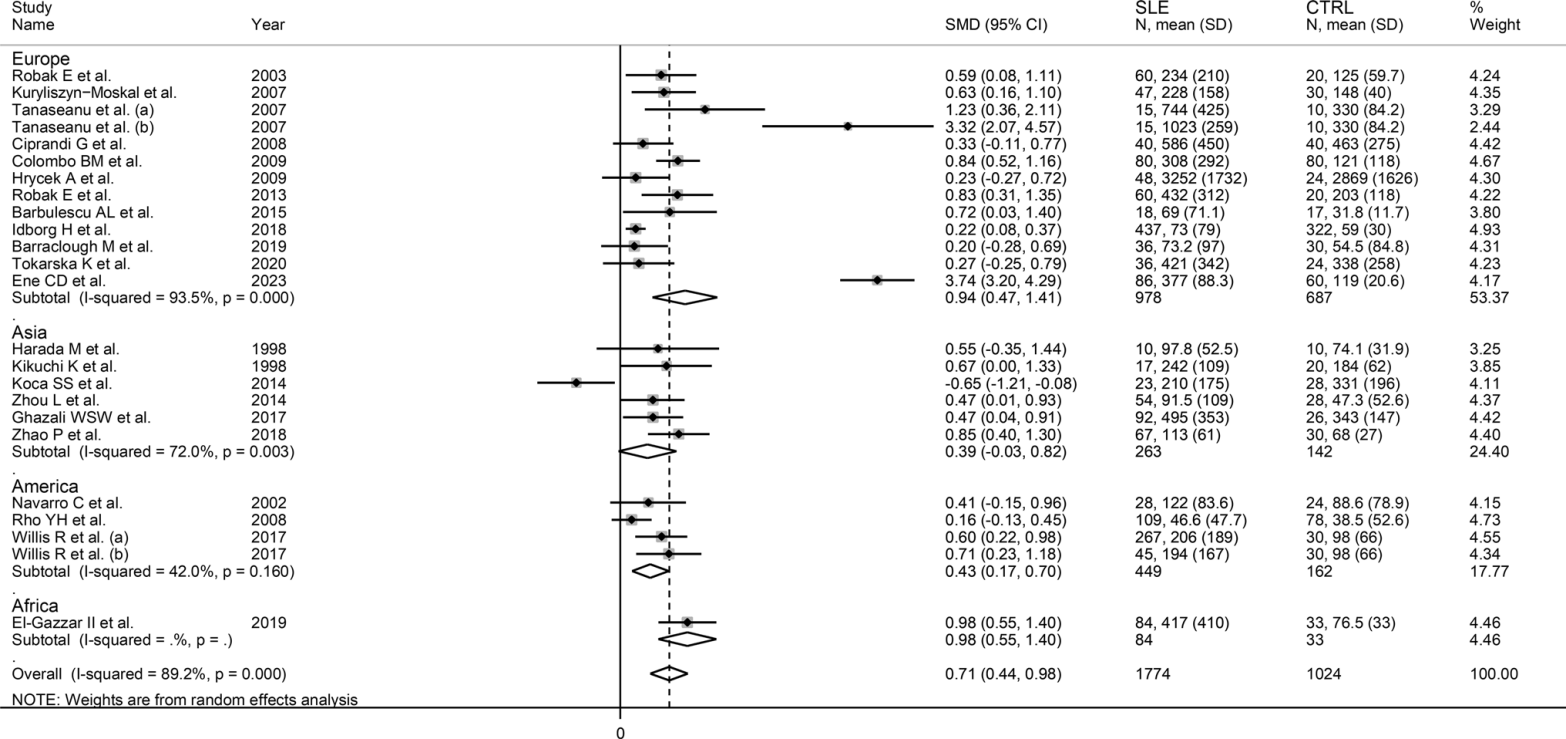
Forest plot of studies investigating vascular endothelial growth factor concentrations in patients with systemic lupus erythematosus and healthy controls according to the geographical area where the study was conducted

**Fig. 7:**
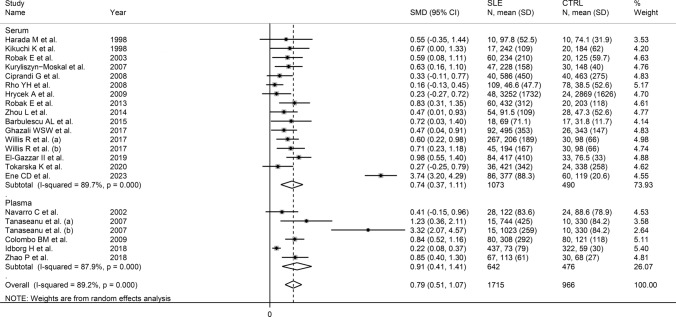
Forest plot of studies investigating vascular endothelial growth factor concentrations in patients with systemic lupus erythematosus and healthy controls according to the matrix type assessed

**Fig. 8:**
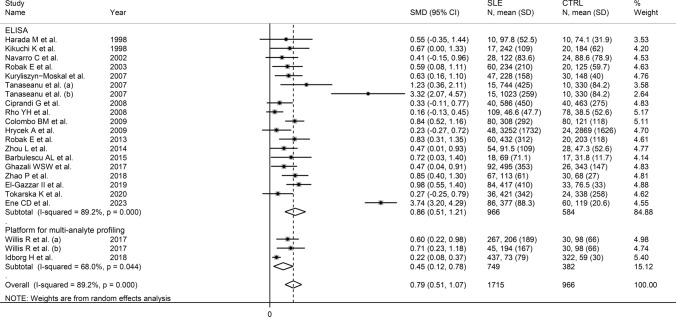
Forest plot of studies investigating vascular endothelial growth factor concentrations in patients with systemic lupus erythematosus and healthy controls according to the type of assay used

Meta-regression analysis did not show any significant associations between the effect size and age (*t* = 0.21, *p* = 0.83), male-to-female ratio (*t* = −0.28, *p* = 0.78), publication year (*t* = 0.73, *p* = 0.47), sample size (*t* = –0.45, *p* = 0.66), mean disease duration (*t* = −0.65, *p* = 0.63), or use of glucocorticoids (*t* = 1.05, *p* = 0.32) and immunosuppressors (*t* = 0.97, *p* = 0.36). Sub-group analysis showed that the pooled SMD was significantly higher in European (SMD = 0.94, 95% CI 0.47 to 1.41, *p* < 0.001; I^2^ = 93.5%, *p* < 0.001) and American studies (SMD = 0.43, 95% CI 0.17 to 0.70, *p* = 0.001; I^2^ = 42.0%, *p* = 0.16) but not Asian studies (SMD = 0.39, 95% CI –0.03 to 0.82, *p* = 0.067; I^2^ = 72.0%, *p* = 0.003; Fig. 6), with lower between-study variance in the American subgroup (I^2^ = 42.0%). There were no significant differences (*p* = 0.55) in pooled SMD between studies assessing serum (SMD = 0.74, 95% CI 0.37 to 1.11, *p* < 0.001; I^2^ = 89.7%, *p* < 0.001) and plasma (SMD = 0.91, 95% CI 0.41 to 1.41, *p* < 0.001; I^2^ = 87.9%, *p* < 0.001; Fig. 7). Furthermore, there were no significant differences (*p* = 0.50) in pooled SMD between studies using an enzyme-linked immunosorbent assay (SMD = 0.86, 95% CI 0.51 to 1.21, *p* < 0.001; I^2^ = 89.2%, *p* < 0.001) and a platform for multi-analyte profiling (SMD = 0.45, 95% CI 0.12 to 0.78, *p* = 0.007; I^2^ = 68.0%, *p* = 0.044; Fig. 8), with lower between-study variance in the latter group.

We maintained the final level of the certainty of evidence as low (level 2) after considering the low-moderate risk of bias in all studies (no change), the high but partially explainable heterogeneity (no change), the lack of indirectness (no change), the moderate effect size (SMD = 0.71; no change) [58], and the presence of publication bias which was addressed using the “trim-and-fill” method (no change).

### Presence of active disease

Six studies (Table 2) investigated VEGF in 477 SLE patients without active disease and 261 with active disease [37, 39, 45, 47, 51, 52]. Four studies were conducted in Europe [37, 39, 45, 52] and two in Asia [47, 51]. An enzyme-linked immunosorbent assay was used to assess serum in all studies except one that used a platform for multi-analyte detection to assess plasma [52]. Disease activity was evaluated by using the Systemic Lupus Erythematosus Disease Activity Index (SLEDAI) in two studies [39, 47, 59], the SLEDAI 2000 (SLEDAI-2 K) in two studies [45, 52, 59], and the scoring system described by of Liang et al. in one study [37, 60]. The remaining study did not provide details regarding the method used to assess disease activity [52]. Four studies had a low risk of bias [45, 47, 51, 52], and two had a moderate risk [37, 39] (Supplementary Table 2).

**Fig. 9:**
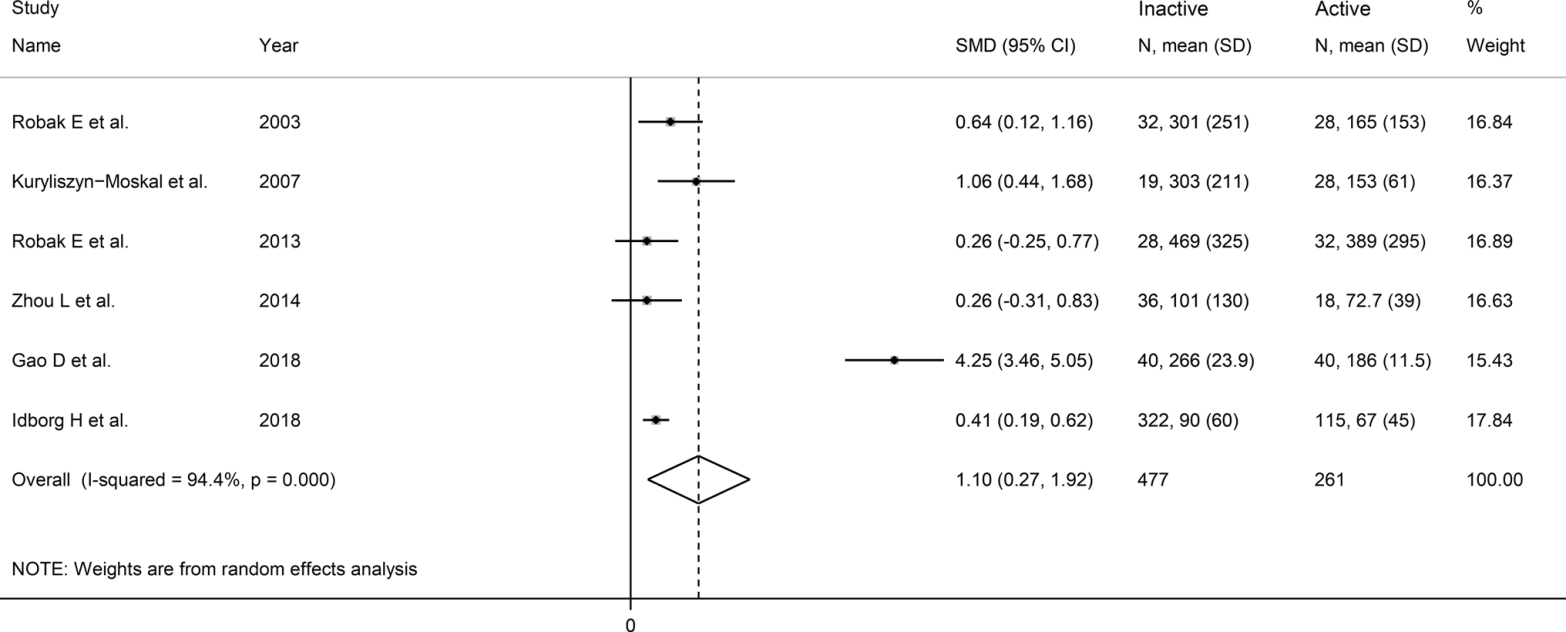
Forest plot of studies investigating vascular endothelial growth factor concentrations in patients with systemic lupus erythematosus with and without active disease

**Fig. 10:**
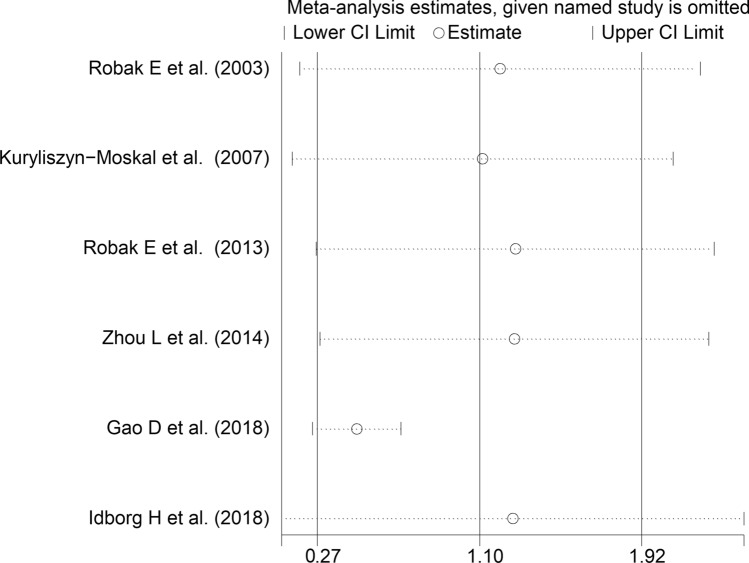
Sensitivity analysis of the association between vascular endothelial growth factor and disease activity

The forest plot (Fig. 9) showed that VEGF concentrations were significantly higher in SLE patients with active disease than those without (SMD = 1.10, 95% CI 0.27 to 1.92, *p* = 0.009; I^2^ = 94.4%, *p* < 0.001). Sensitivity analysis (Fig. 10) showed a tangible effect of the study by Gao et al. on the pooled SMD [51]. After removing this study, the effect size was reduced yet still significant (SMD = 0.47, 95% CI 0.25 to 0.70, *p* < 0.001). Furthermore, the heterogeneity was substantially lower (I^2^ = 24.9%, *p* = 0.25). We downgraded the final level of the certainty of evidence to very low (level 1) as the limited number of studies prevented assessing publication bias and conducting meta-regression and sub-group analyses.

### Presence of lupus nephritis

Four studies (Table 3) investigated VEGF in 331 SLE patients with lupus nephritis and 424 without [49, 52, 53, 57]. Two studies were conducted in Europe [52, 57] and two in Asia [49, 53]. VEGF was measured using an enzyme-linked immunosorbent assay in three studies [49, 53, 57] and a platform for multi-analyte profiling in one [52]. Serum was assessed in two studies [49, 57] and plasma in the other two [52, 53]. All studies had a low risk of bias (Supplementary Table 2).

**Fig. 11:**
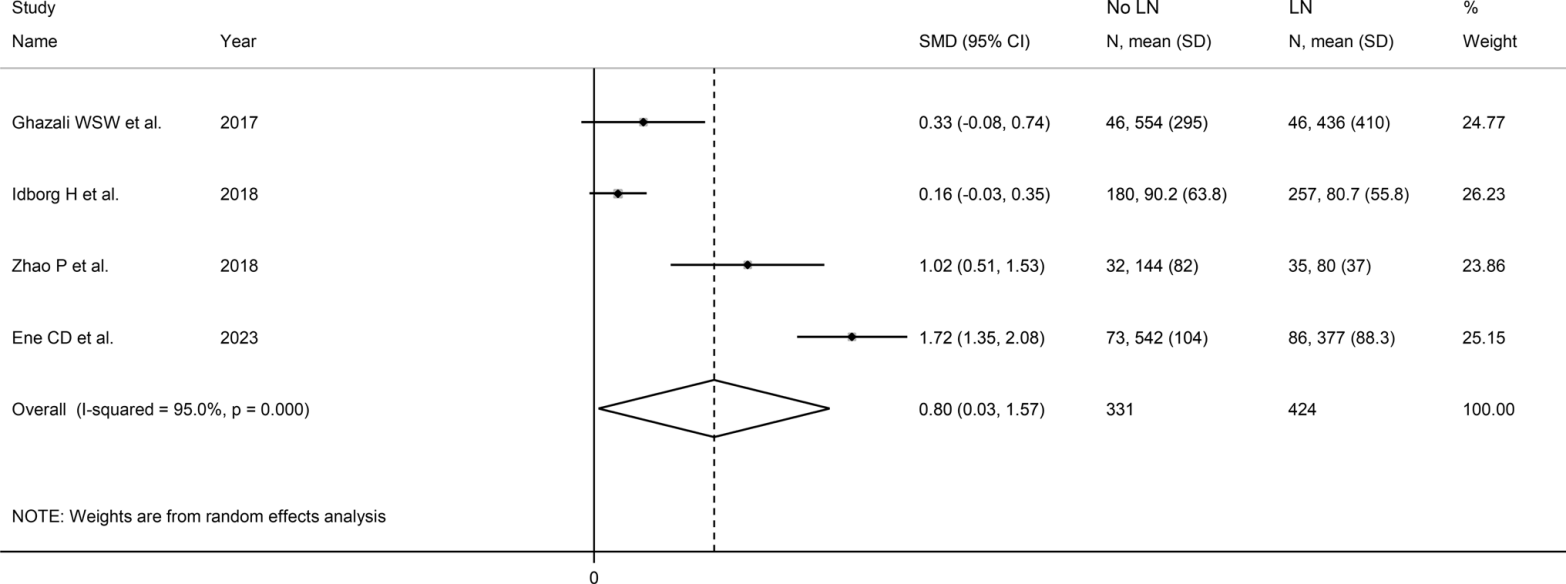
Forest plot of studies investigating vascular endothelial growth factor concentrations in patients with systemic lupus erythematosus with and without lupus nephritis

The forest plot (Fig. 11) showed that SLE patients with lupus nephritis had significantly higher VEGF concentrations than those without (SMD = 0.80, 95% CI 0.03 to 1.57, *p* = 0.042, I^2^ = 95.0%, *p* < 0.001). We downgraded the final level of the certainty of evidence to very low (level 1) as the limited number of studies prevented sensitivity analysis, assessing publication bias, and conducting meta-regression and sub-group analyses.

### Presence of pulmonary arterial hypertension

One European study with a moderate risk of bias investigated plasma VEGF in 30 patients, 15 with pulmonary arterial hypertension and 15 without [40]. VEGF concentrations were significantly higher in patients with pulmonary arterial hypertension (1023.1 ± 259.07 vs. 744.2 ± 425.1 pg/mL, *p* < 0.05).

## Discussion

In contrast to the results of in vitro studies, generally reporting a reduced VEGF expression in SLE [19–21], this systematic review and meta-analysis has shown that SLE patients have significantly higher circulating VEGF concentrations when compared to healthy controls. VEGF concentrations were further elevated in those SLE patients with active disease and specific complications, e.g. lupus nephritis. Possible reasons for the discrepancy between in vitro and in vivo studies include investigating VEGF gene expression vs. VEGF concentrations, the relatively limited number and types of cells studied in vitro, and the various sources of VEGF potentially contributing to its circulating concentrations in vivo. Although a significant heterogeneity was observed in the main analyses, it is noteworthy that virtually all selected studies reported circulating VEGF elevations (21 out of 22 studies investigating SLE presence, Fig. 2; six out of six studies investigating active disease, Fig. 9; four out of four studies investigating lupus nephritis, Fig. 11). However, there was limited evidence regarding possible associations with other complications, with only one study investigating pulmonary arterial hypertension. We could only conduct meta-regression and subgroup analyses in studies investigating VEGF in SLE patients and controls. Such analyses showed no significant associations between the effect size and various study and patient characteristics, mainly mean disease duration, use of glucocorticoids or immunosuppressors, biological matrix assessed, and analytical method used. By contrast, there were significant SLE-associated VEGF elevations in European and American studies but not in Asian studies. The lack of associations observed with disease duration suggests that VEGF elevations are also evident during the early stages of the disease.

Although in vitro and in vivo studies have provided contrasting results regarding the possible link between VEGF and SLE, several lines of evidence support the upregulation of VEGF in this patient group. For example, the presence of pro-inflammatory and hypoxic states, a common feature in atherosclerosis [14, 61], is well known to upregulate the hypoxia-inducible factor 1 subunit alfa (HIF-1α) in endothelial cells [62]. The consequent HIF-1α-mediated upregulation of VEGF can be considered a compensatory mechanism to ensure the structural and functional integrity of the endothelium in the presence of atherogenic insults [63]. However, there is also evidence that VEGF might exert detrimental effects on atherosclerosis. For example, studies have reported that VEGF can suppress repair mechanisms in endothelial cells, with the consequent stimulation of monocyte adhesion and transmigration into the intima-media layer of the arterial wall, activation of vascular smooth muscle cells, and initiation of the atherosclerotic process [63]. The pro-angiogenic effects of VEGF can exert additional detrimental effects on the stability of the atherosclerotic plaque by promoting local neovascularization, with a consequent increased risk of plaque rupture and thrombus formation [64]. Therefore, the previously reported elevations of VEGF and pro-inflammatory cytokines in SLE patients also align with the increased risk of atherosclerosis and cardiovascular disease in this group [4–7, 13, 22]. Pending the results of additional studies, the association between VEGF and inflammation can also explain, at least partly, the further elevations in VEGF concentrations observed in SLE patients with active disease and lupus nephritis. Both these conditions are characterised by a particularly pronounced pro-inflammatory state. Most of the clinical manifestations of active disease reflected in validated tools such as the British Isles Lupus Activity Group (BILAG) score, the European Consensus Lupus Activity Measure (ECLAM), the SLE Index Score (SIS), the SLE Disease Activity Index (SLEDAI) and the Systemic Lupus Erythematosus Measure (SLAM) indicate the presence of excess inflammation in individual organs or the elevations of conventional inflammatory biomarkers, e.g. erythrocyte sedimentation rate [65]. Excess inflammation has also been documented in lupus nephritis. In a recent study, SLE patients with lupus nephritis were shown to have significant elevations in haematological indices of inflammation, i.e. neutrophil-to-lymphocyte ratio, platelet-to-lymphocyte ratio, systemic inflammatory index, and systemic inflammatory response index, when compared to SLE patients without lupus nephritis [66]. Similar observations have been reported using other biomarkers of inflammation and immune activation [67, 68].

One interesting finding in our subgroup analysis was the significant difference in the effect size of VEGF elevations in SLE patients vs. healthy controls according to the study’s geographical location. Such differences may reflect geographical differences in SLE prevalence, clinical characteristics, and VEGF expression. It is well known that non-Caucasian populations have a higher risk of SLE. Furthermore, in these groups, the disease often presents acutely, with more severe clinical manifestations and organ involvement [69–73]. Studies have also reported opposite associations between specific VEGF polymorphisms and autoimmune diseases in Asians vs. other populations [74–78]. Further research should investigate the possible role of geographical factors in mediating the association between VEGF and SLE as well as disease activity and specific organ dysfunction.

This systematic review and meta-analysis has several strengths, e.g. the assessment of VEGF in SLE patients and different subgroups (active disease and presence of specific complications), the assessment of the certainty of evidence for each available endpoint, and the evaluation of associations between the effect size and specific study and patient variables. Important limitations include the limited number of studies in patients with active disease and individual complications and the heterogeneity observed. However, the latter could be partially explained in studies between SLE patients and controls by geographical location and analytical method used for measuring VEGF.

In conclusion, our study has shown significant elevations in VEGF concentrations in SLE patients overall and in those with active disease and lupus nephritis. Further research is warranted to confirm our findings and investigate a wide range of SLE subtypes in different continents to further support the role of VEGF as a candidate biomarker in this patient group.

## Supplementary Information

Below is the link to the electronic supplementary material.

Supplementary file1 (DOCX 32 kb)

Supplementary file2 (DOCX 47 kb)

## Data Availability

The data that support the findings of this systematic review and meta-analysis are available from AZ upon reasonable request.
