# Supplementary material for: The vascular endothelial growth factor as a candidate biomarker of systemic lupus erythematosus: a GRADE-assessed systematic review and meta-analysis
Source: Clin Exp Med. 2024 Sep 11;24(1):218. doi: 10.1007/s10238-024-01487-w (PMC11390800; doi:10.1007/s10238-024-01487-w)
Supplement: Supplementary file 2 — Supplementary file2 (DOCX 47 kb) [file 10238_2024_1487_MOESM2_ESM.docx]

**Supplementary Table 2.** Assessment of the risk of bias using the Joanna Briggs Institute critical appraisal checklist.

| **Study** | **Were the inclusion criteria clearly defined?** | **Were the subjects and the setting described in detail?** | **Was the exposure measured in a reliable way?** | **Were standard criteria used to assess the condition?** | **Were confounding factors identified?** | **Were strategies to deal with confounding factors stated?** | **Were the outcomes measured in a reliable way?** | **Was appropriate statistical analysis used?** | **Risk of bias** |
| --- | --- | --- | --- | --- | --- | --- | --- | --- | --- |
| Harada M et al. [1] | No | Yes | Yes | Yes | No | No | Yes | Yes | Moderate |
| Kikuchi K et al. [2] | Yes | Yes | Yes | No | No | No | Yes | Yes | Moderate |
| Robak E et al. [3] | Yes | Yes | Yes | Yes | No | No | Yes | Yes | Low |
| Navarro C et al. [4] | No | Yes | Yes | Yes | No | No | Yes | Yes | Moderate |
| Kuryliszyn−Moskal et al. [5] | No | Yes | Yes | Yes | No | No | Yes | Yes | Moderate |
| Tanaseanu et al. [6] | No | Yes | Yes | No | No | No | Yes | Yes | Moderate |
| Ciprandi G et al. [7] | No | Yes | Yes | Yes | No | No | Yes | Yes | Moderate |
| Rho YH et al. [8] | Yes | Yes | Yes | Yes | Yes | Yes | Yes | Yes | Low |
| Colombo BM et al. [9] | Yes | Yes | Yes | Yes | Yes | Yes | Yes | Yes | Low |
| Hrycek A et al. [10] | No | Yes | Yes | Yes | No | No | Yes | Yes | Moderate |
| Robak E et al. [11] | No | Yes | Yes | Yes | No | No | Yes | Yes | Moderate |
| Koca SS et al. [12] | No | Yes | Yes | Yes | No | No | Yes | Yes | Moderate |
| Zhou L et al. [13] | Yes | Yes | Yes | Yes | No | No | Yes | Yes | Low |
| Barbulescu AL et al. [14] | Yes | Yes | Yes | Yes | No | No | Yes | Yes | Low |
| Ghazali WSW et al. [15] | Yes | Yes | Yes | Yes | No | No | Yes | Yes | Low |
| Willis R et al. [16] | Yes | Yes | Yes | Yes | Yes | Yes | Yes | Yes | Low |
| Gao D et al. [17] | Yes | Yes | Yes | Yes | No | No | Yes | Yes | Low |
| Idborg H et al. [18] | Yes | Yes | Yes | Yes | No | No | Yes | Yes | Low |
| Zhao P et al. [19] | Yes | Yes | Yes | Yes | No | No | Yes | Yes | Low |
| El-Gazzar II et al. [20] | No | Yes | Yes | Yes | Yes | Yes | Yes | Yes | Low |
| Barraclough M et al. [21] | Yes | Yes | Yes | Yes | No | No | Yes | Yes | Low |
| Tokarska K et al. 2020, [22] | Yes | Yes | Yes | Yes | No | No | Yes | Yes | Low |
| Ene CD et al. [23] | Yes | Yes | Yes | Yes | No | No | Yes | Yes | Low |

**References**

1. Harada M, Mitsuyama K, Yoshida H, et al. Vascular endothelial growth factor in patients with rheumatoid arthritis. Scand J Rheumatol 1998;27(5):377-80. doi: 10.1080/03009749850154429

2. Kikuchi K, Kubo M, Kadono T, Yazawa N, Ihn H, Tamaki K. Serum concentrations of vascular endothelial growth factor in collagen diseases. Br J Dermatol 1998;139(6):1049-51. doi: 10.1046/j.1365-2133.1998.02563.x

3. Robak E, Sysa-jędrzejewska A, Robak T. Vascular endothelial growth factor and its soluble receptors VEGFR‐1 and VEGFR‐2 in the serum of patients with systemic lupus erythematosus. Mediators of Inflammation 2000;12(5):293-8. doi: 10.1080/09629350310001619726

4. Navarro C, Candia-Zuniga L, Silveira LH, et al. Vascular endothelial growth factor plasma levels in patients with systemic lupus erythematosus and primary antiphospholipid syndrome. Lupus 2002;11(1):21-4. doi: 10.1191/0961203302lu131oa

5. Kuryliszyn-Moskal A, Klimiuk PA, Sierakowski S, Ciolkiewicz M. Vascular endothelial growth factor in systemic lupus erythematosus: relationship to disease activity, systemic organ manifestation, and nailfold capillaroscopic abnormalities. Arch Immunol Ther Exp (Warsz) 2007;55(3):179-85. doi: 10.1007/s00005-007-0017-7

6. Tanaseanu C, Tudor S, Tamsulea I, Marta D, Manea G, Moldoveanu E. Vascular endothelial growth factor, lipoporotein-associated phospholipase A2, sP-selectin and antiphospholipid antibodies, biological markers with prognostic value in pulmonary hypertension associated with chronic obstructive pulmonary disease and systemic lupus erithematosus. Eur J Med Res 2007;12(4):145-51. doi:

7. Ciprandi G, Murdaca G, Colombo BM, De Amici M, Marseglia GL. Serum vascular endothelial growth factor in allergic rhinitis and systemic lupus erythematosus. Hum Immunol 2008;69(8):510-2. doi: 10.1016/j.humimm.2008.05.010

8. Rho YH, Chung CP, Oeser A, et al. Novel cardiovascular risk factors in premature coronary atherosclerosis associated with systemic lupus erythematosus. J Rheumatol 2008;35(9):1789-94. doi:

9. Colombo BM, Cacciapaglia F, Puntoni M, et al. Traditional and non traditional risk factors in accelerated atherosclerosis in systemic lupus erythematosus: role of vascular endothelial growth factor (VEGATS Study). Autoimmun Rev 2009;8(4):309-15. doi: 10.1016/j.autrev.2008.10.002

10. Hrycek A, Janowska J, Cieslik P. Selected angiogenic cytokines in systemic lupus erythematosus patients. Autoimmunity 2009;42(5):459-66. doi: 10.1080/08916930902960339

11. Robak E, Kulczycka-Siennicka L, Gerlicz Z, Kierstan M, Korycka-Wolowiec A, Sysa-Jedrzejowska A. Correlations between concentrations of interleukin (IL)-17A, IL-17B and IL-17F, and endothelial cells and proangiogenic cytokines in systemic lupus erythematosus patients. European Cytokine Network 2013;24(1):60-8. doi: 10.1684/ecn.2013.0330

12. Koca SS, Akbas F, Ozgen M, et al. Serum galectin-3 level in systemic sclerosis. Clin Rheumatol 2014;33(2):215-20. doi: 10.1007/s10067-013-2346-8

13. Zhou L, Lu G, Shen L, Wang L, Wang M. Serum Levels of Three Angiogenic Factors in Systemic Lupus Erythematosus and Their Clinical Significance. BioMed Research International 2014;2014:1-6. doi: 10.1155/2014/627126

14. Barbulescu AL, Vreju AF, Buga AM, et al. Vascular endothelial growth factor in systemic lupus erythematosus - correlations with disease activity and nailfold capillaroscopy changes. Rom J Morphol Embryol 2015;56(3):1011-6. doi:

15. Wan Ghazali WS, Iberahim R, Mohd Ashari NS. Serum Vascular Endothelial Growth Factor (VEGF) as a Biomarker for Disease Activity in Lupus Nephritis. Malaysian Journal of Medical Sciences 2017;24(5):62-72. doi: 10.21315/mjms2017.24.5.7

16. Willis R, Smikle M, DeCeulaer K, et al. Clinical associations of proinflammatory cytokines, oxidative biomarkers and vitamin D levels in systemic lupus erythematosus. Lupus 2017;26(14):1517-27. doi: 10.1177/0961203317706557

17. Gao D, Shao J, Jin W, Xia X, Qu Y. Correlations of serum cystatin C and hs-CRP with vascular endothelial cell injury in patients with systemic lupus erythematosus. Panminerva Med 2018;60(4):151-5. doi: 10.23736/S0031-0808.18.03466-3

18. Idborg H, Eketjäll S, Pettersson S, et al. TNF-α and plasma albumin as biomarkers of disease activity in systemic lupus erythematosus. Lupus Science & Medicine 2018;5(1). doi: 10.1136/lupus-2018-000260

19. Zhao P, Miao J, Zhang K, Lv M, Han Q, Zhu P. Circulating Angiogenic T Cells Are Increased in Lupus Nephritis Patients. Med Sci Monit 2018;24:5384-90. doi: 10.12659/MSM.908406

20. El-Gazzar II, Ibrahim SE, El-Sawy WS, Fathi HM, Eissa AH. Assessment of vascular endothelial growth factor in systemic lupus erythematosus patients with anti-phospholipid syndrome. The Egyptian Rheumatologist 2019;41(1):41-5. doi: 10.1016/j.ejr.2018.03.005

21. Barraclough M, McKie S, Parker B, et al. Altered cognitive function in systemic lupus erythematosus and associations with inflammation and functional and structural brain changes. Ann Rheum Dis 2019;78(7):934-40. doi: 10.1136/annrheumdis-2018-214677

22. Tokarska K, Bogaczewicz J, Robak E, Wozniacka A. The role of endocan and selected pro-inflammatory cytokines in systemic lupus erythematosus. Postepy Dermatol Alergol 2020;37(6):898-903. doi: 10.5114/ada.2019.90060

23. Ene CD, Nicolae I. The Inflammatory Profile Orchestrated by Inducible Nitric Oxide Synthase in Systemic Lupus Erythematosus. J Pers Med 2023;13(6). doi: 10.3390/jpm13060934
